# Towel Uterus Model for Uterine Compression Sutures Technical Skills Training: A Review of Literature and Development of a Performance Rubric

**DOI:** 10.7759/cureus.2725

**Published:** 2018-06-01

**Authors:** Milena Garofalo, Glenn D Posner

**Affiliations:** 1 Department of Obstetrics and Gynaecology, Faculty of Medicine, Mcgill University, Montreal, Qc, Canada, McGill University Health Centre; 2 Department of Innovation in Medical Education, University of Ottawa

**Keywords:** simulator, uterine compression sutures, obstetrics, medical education, simulation-based assessment

## Abstract

Postpartum hemorrhage (PPH) continues to be the leading cause of maternal mortality worldwide, occurring in about five percent of deliveries. The most common cause of PPH is uterine atony, and a number of medical and surgical management techniques are available to prevent morbidity and mortality associated with PPH in this context. Uterine compression sutures provide a more conservative surgical approach, allowing for the preservation of fertility. Obstetrics and Gynecology (Ob/Gyn) residents need to be adequately trained to competently perform this technique.

The goal of this surgical skills training is for Ob/Gyn residents to be able to surgically manage PPH using uterine compression sutures. A uterine towel model for surgical skills training in the use of uterine compression sutures was developed. The simulator is explained and compared to similar models. Possible ways to implement and use the simulator in a simulation curriculum are also described. A performance-based assessment rubric was also developed in order to formatively aid with the learning and understanding of the technique. Much work is still needed to test the validity and reliability of this tool, but based on current literature, results may be promising.

## Introduction and background

The problem and need

Postpartum hemorrhage (PPH) continues to be the leading cause of maternal mortality worldwide, occurring in about five percent of deliveries. An estimated 140,000 women die each year secondary to PPH. It is a preventable medical emergency that requires a timely response. The most common cause of PPH is uterine atony, i.e., the inability of the uterine musculature to adequately contract, thus leading to excess blood loss. Most cases of PPH secondary to uterine atony are managed successfully with medical treatment, such as oxytocin or misoprostol. However, occasionally after a vaginal delivery, and more frequently during a cesarean delivery, a surgical approach may be needed. Surgical management of PPH includes balloon tamponade, uterine artery embolization (by interventional radiology), internal iliac or uterine artery ligation, uterine compression sutures, and ultimately hysterectomy [1-2]. Technical skills are required in order to perform these surgical procedures; and as they are relatively rare, Obstetrics and Gynecology (Ob/Gyn) residents, both senior and junior alike, are rarely exposed to them. When they are exposed, the patient is usually unstable and has already had a large amount of blood loss; thus, it is dangerous and unsafe for the patient in question to have someone inexperienced practice on them. The Royal College “Objectives of Training” for Ob/Gyn state that extensive knowledge is required for the “etiology and management, medical and surgical, of early and delayed postpartum hemorrhage” and that a fully trained resident must be competent to perform independently both “nonsurgical and surgical management of moderate and severe postpartum hemorrhage.” This includes the use of uterine compression sutures [3]. This novel simulator described here focuses on the technical skills required to perform the three most common uterine compression sutures, namely the B-Lynch, Hayman, and Cho sutures. 

**Figure 1:**
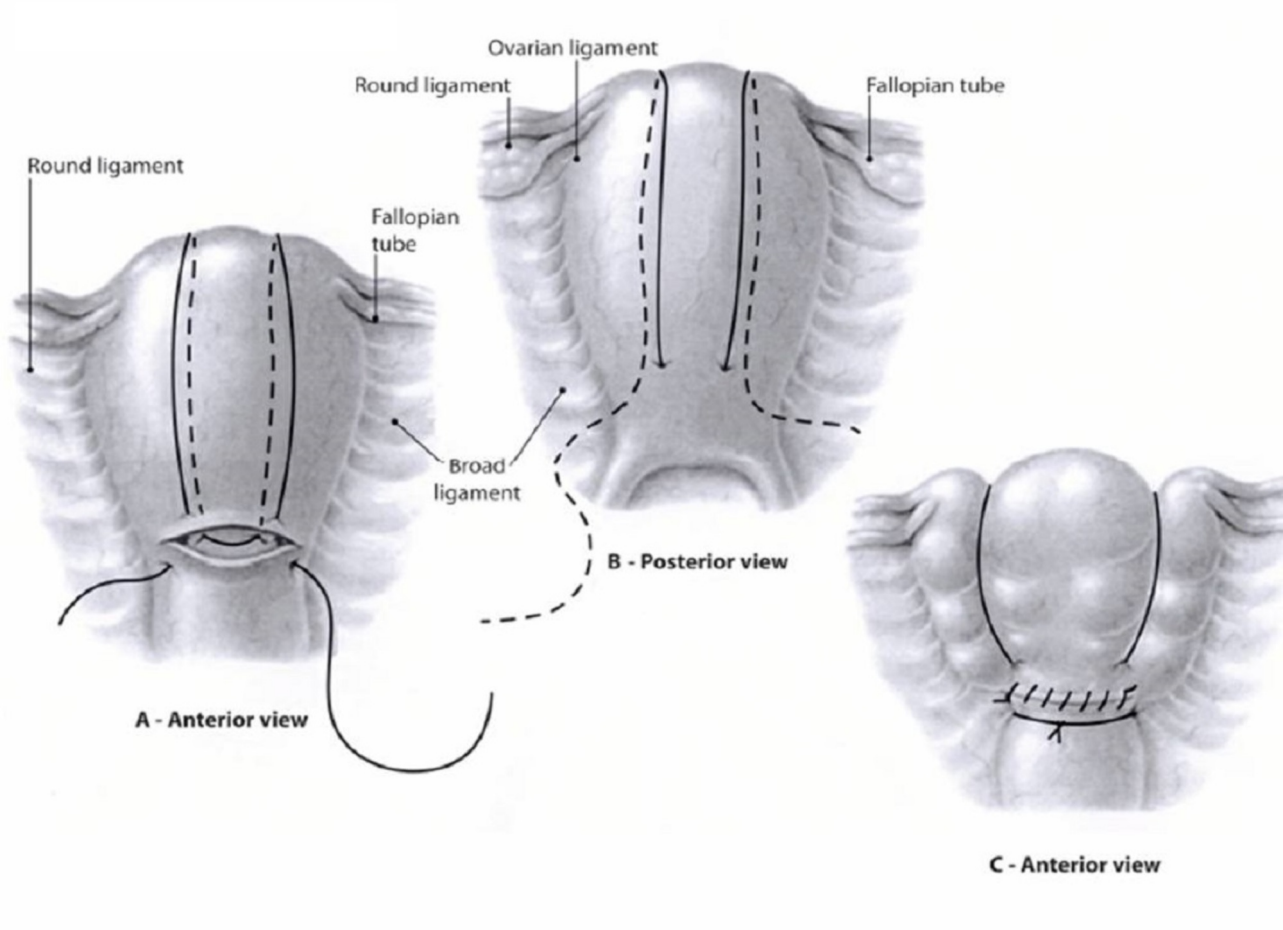
B-Lynch compression suture [2].

**Figure 2:**
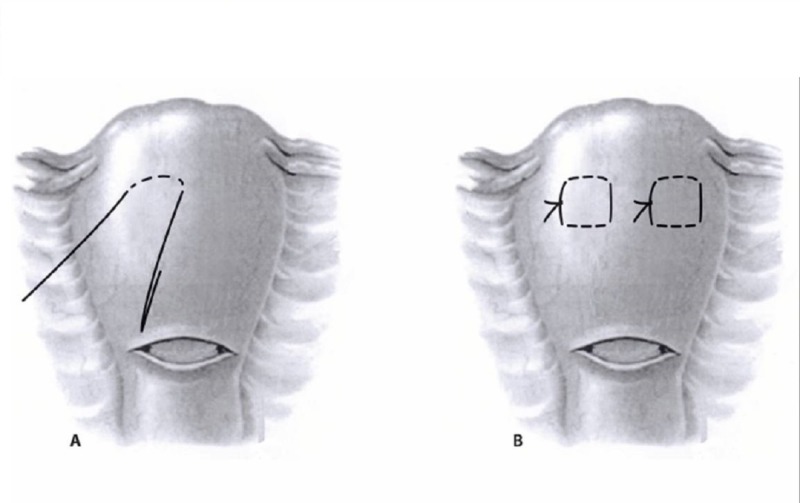
Cho compression suture [2].

**Figure 3:**
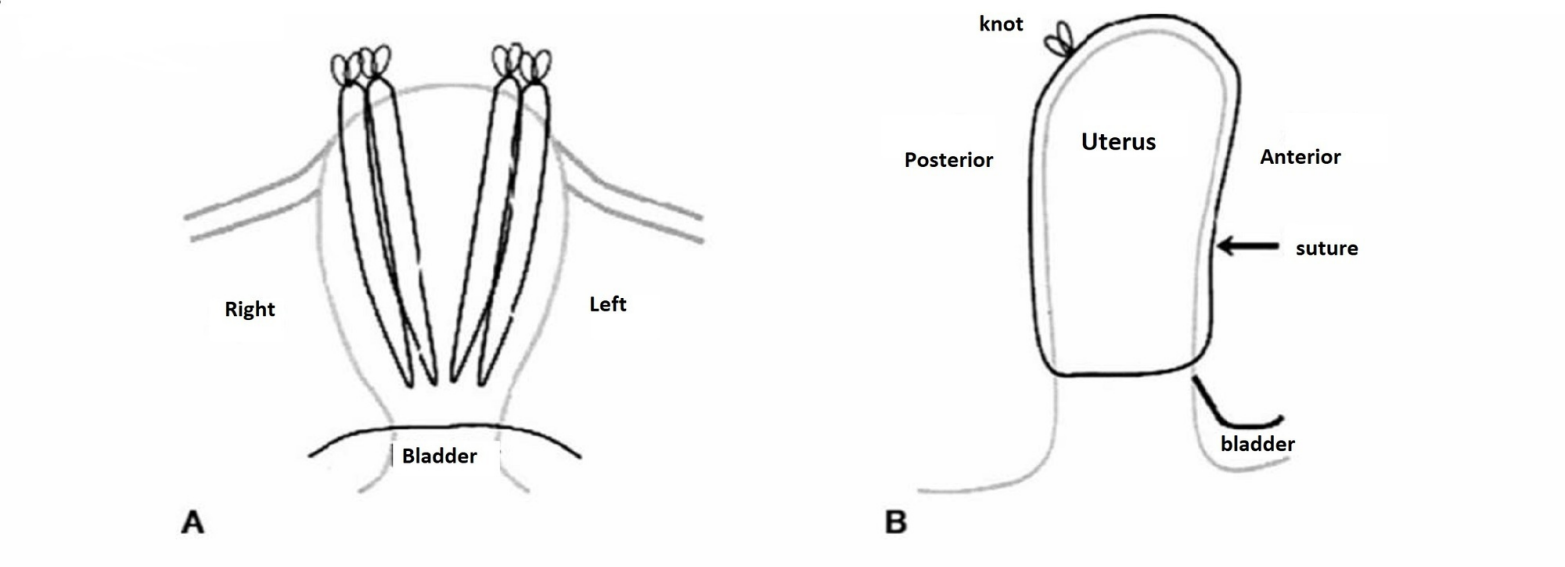
Hayman compression suture [6].

The idea of uterine compression sutures was first introduced by Christopher B. Lynch in 1997 [4]. Since then, various compression sutures, named after their inventors, have been described in observational studies and case series. No higher-level evidence has demonstrated whether compression sutures are better and safer for achieving hemostasis in PPH than other methods, or whether one suture type is better or safer than the other. Other compression sutures include the Cho suture described in 2000 and the Hayman suture (also called modified B-Lynch) described in 2002 [5-6] (see Figures 1-3 for illustrations of these three suture types). They have the advantage of not only being potentially life-saving, but they all also allow for preservation of the uterus and fertility. In the past nine years, there have been several reviews published on the subject comparing the different uterine compression sutures [7-10]. A 2007 systematic review reported a 91.7% success rate for various compression sutures [7]. Most published data discuss the original B-Lynch suture, whose success rate is considered to be 99.5%, based on personal communication with Dr. B. Lynch [10]. There appears to be a substantially decreased success rate, 75%, with a delay of two to six hours following the delivery [8]. Compared to the other types, the B-Lynch suture has the advantage that it does not sew the anterior and posterior uterine walls together, thus potentially decreasing the risk of pyometra and synechiae. These two complications have been reported with the Cho suture [10]. The Hayman technique is a simple modification of the B-Lynch, which does not require the opening of the uterine cavity (hysterotomy). It is thus simpler and quicker. For all of these techniques, data on future fertility are scarce [9].

## Review

Uterine compression sutures technical skills training

*Goals and Objectives*: The goal of this surgical skills training is for Ob/Gyn residents to be able to surgically manage PPH with the use of uterine compression sutures. There are six main objectives to the training which are included in the more detailed performance rubric at the end of the paper and which form the criteria of the assessment tool. At the end of the session, the resident should be able to:

1.      Recognize when surgical management of PPH is needed.

2.      Know the different options for surgical management of PPH, including the relevant anatomy.

3.      Describe and compare B-Lynch, Hayman, and Cho uterine compression sutures.

4.      Select the appropriate suture/needle material and instruments for the compression suture.

5.      Demonstrate B-Lynch, Hayman, and Cho uterine compression sutures on a towel uterine model (the simulator).

6.      Perform the compression sutures in a timely and efficient manner.

Educational tool: the simulator

**Figure 4:**
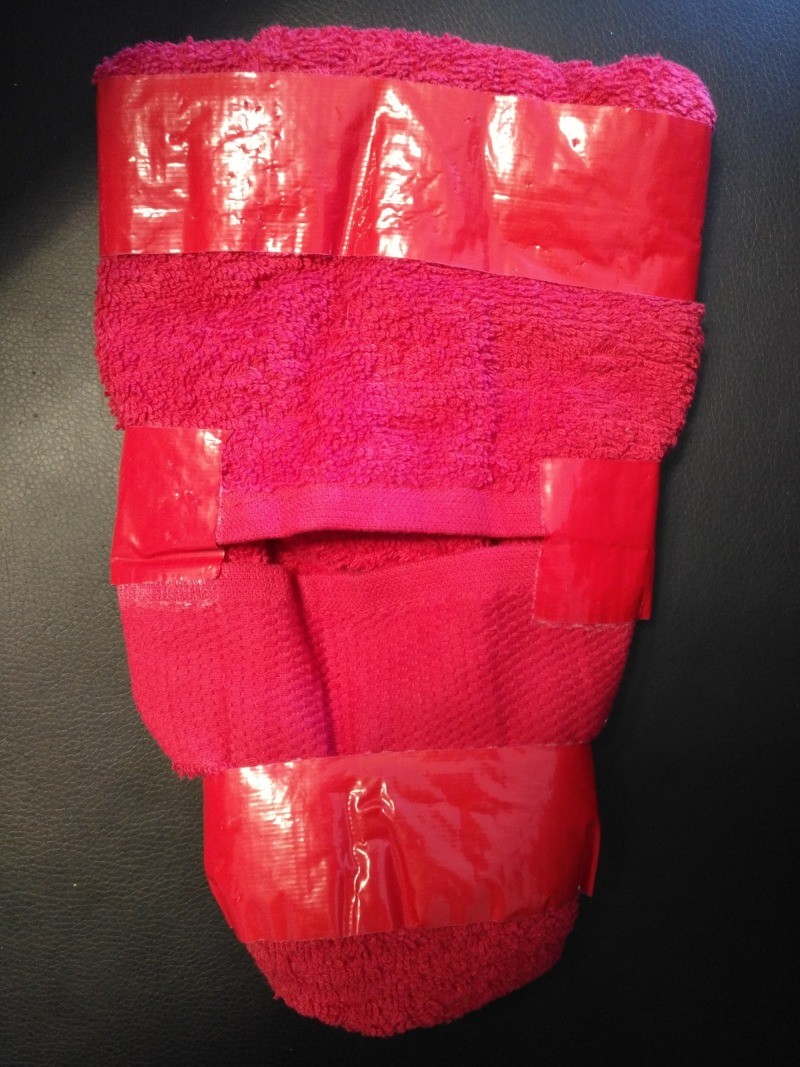
Uterine towel model - our simulator

**Figure 5:**
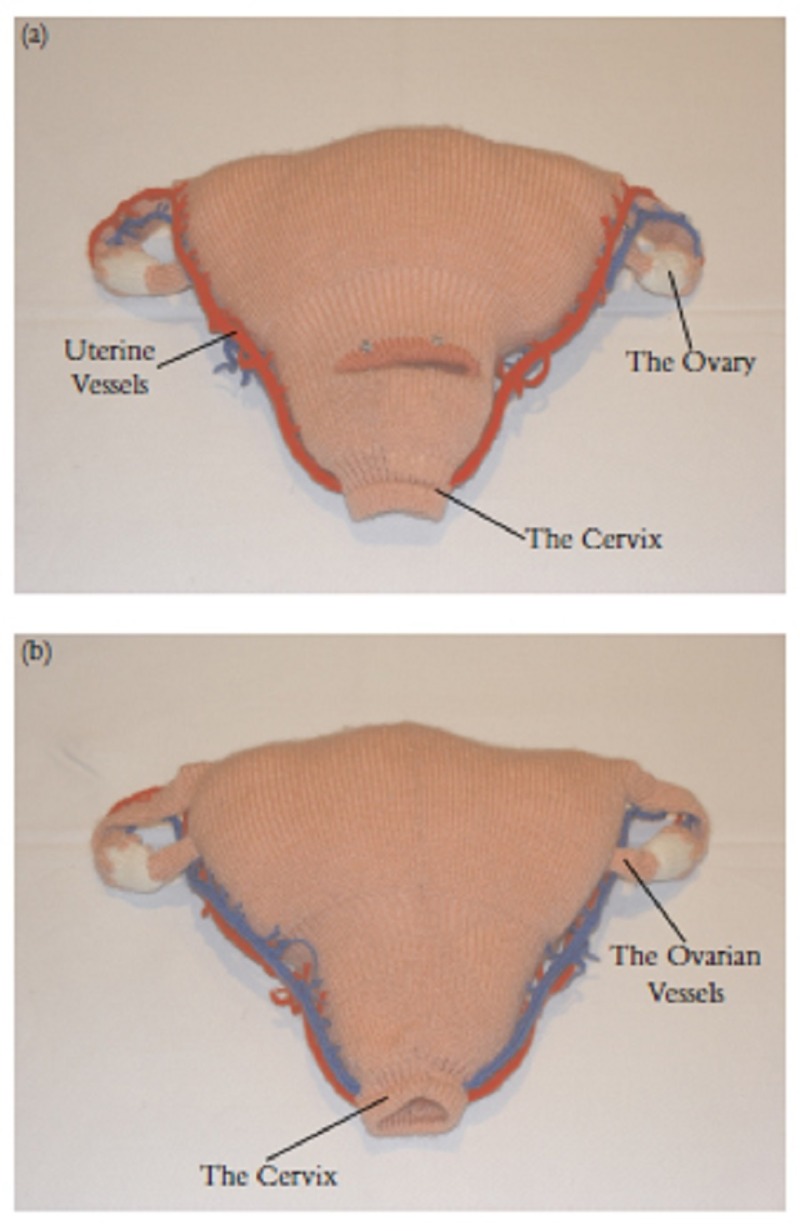
Knitted wool model [11].

The task trainer/simulator developed and described for this project is shown in Figure 4. It was made using a medium-sized red towel, folded in such a way so as to simulate the hysterotomy (lower transverse uterine incision), with the two ends of the towel. Red tape was used to create the shape of the uterus and to hold the model together. In order to increase the realism of the model, the uterine vessels, fallopian tubes, ovaries, and broad ligament may also be included as in the models in Figures 5, 7, 9. This model is feasible due to its low cost (approximately CAD 5 per model), and just like the other similar models described below, it has both instrumental and procedural fidelity. This is because the same instruments, materials, and procedural steps as used in real-life can be replicated with the simulator possibly leading to the transfer of skills to the real world. It does not provide tissue or physiologic fidelity. Anatomic fidelity may be increased by adding the uterine arteries, fallopian tubes, and ovaries.

**Figure 6:**
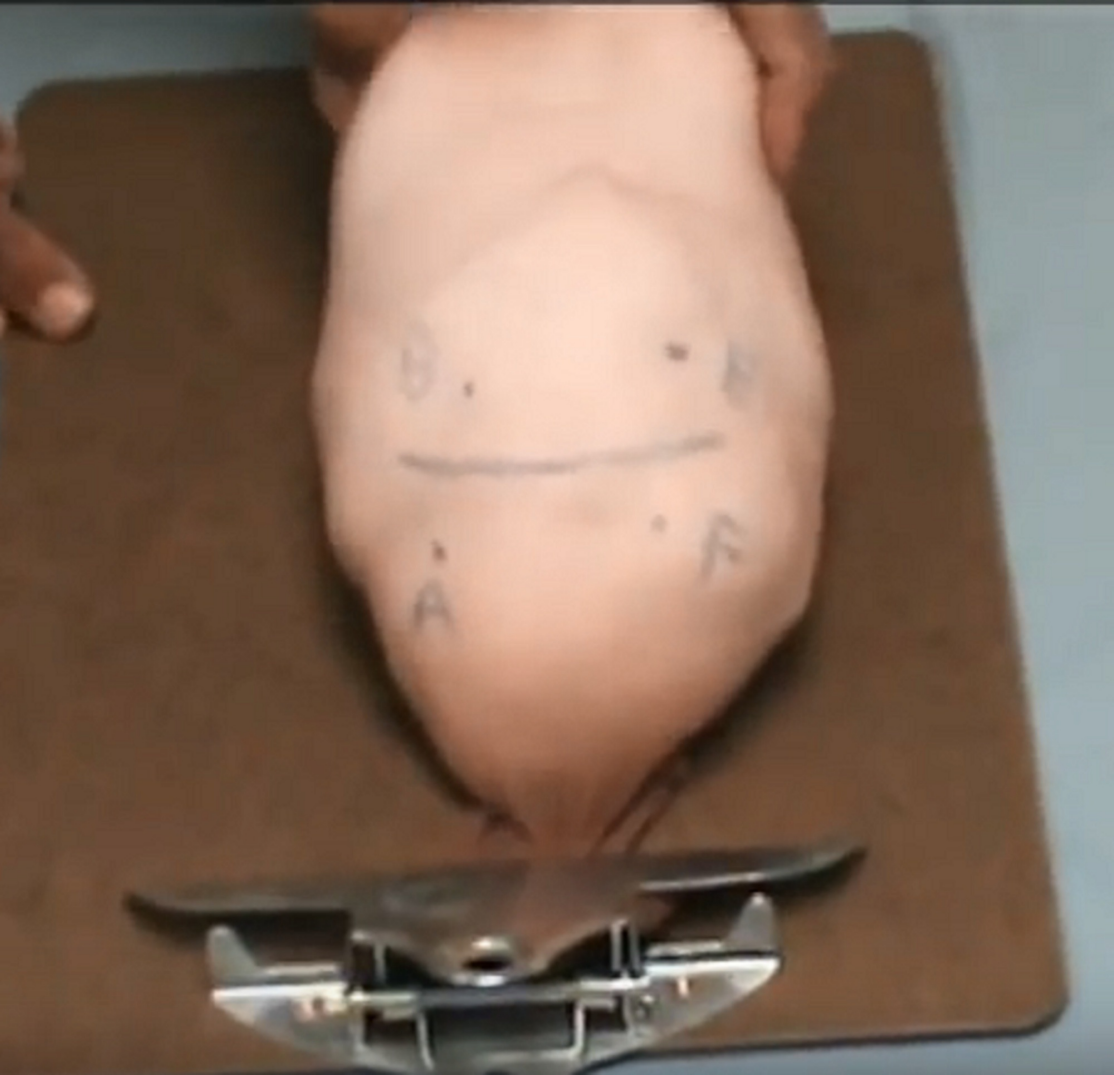
Filled pantyhose stocking model [12].

**Figure 7:**
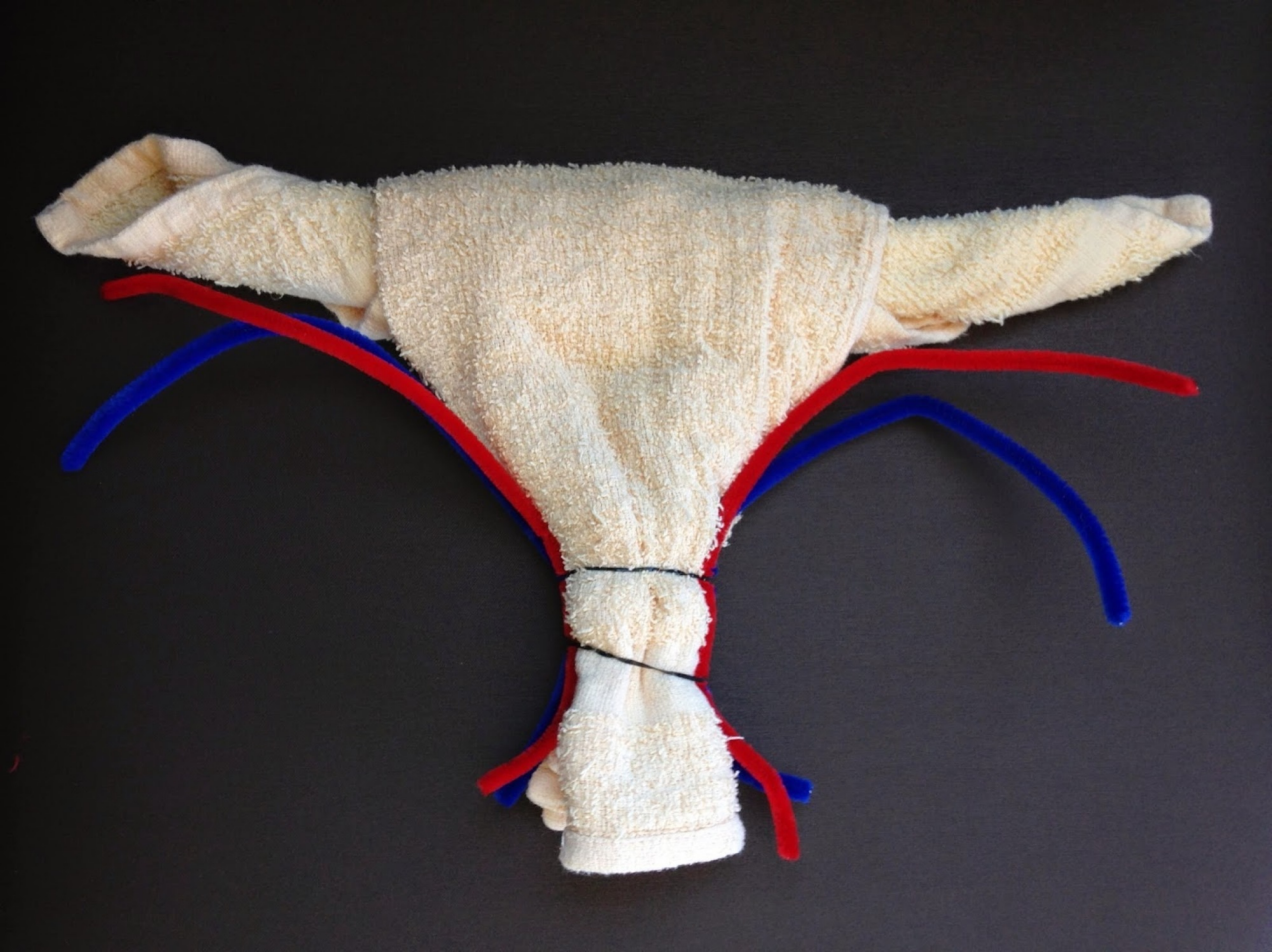
Face cloth model [13].

**Figure 8:**
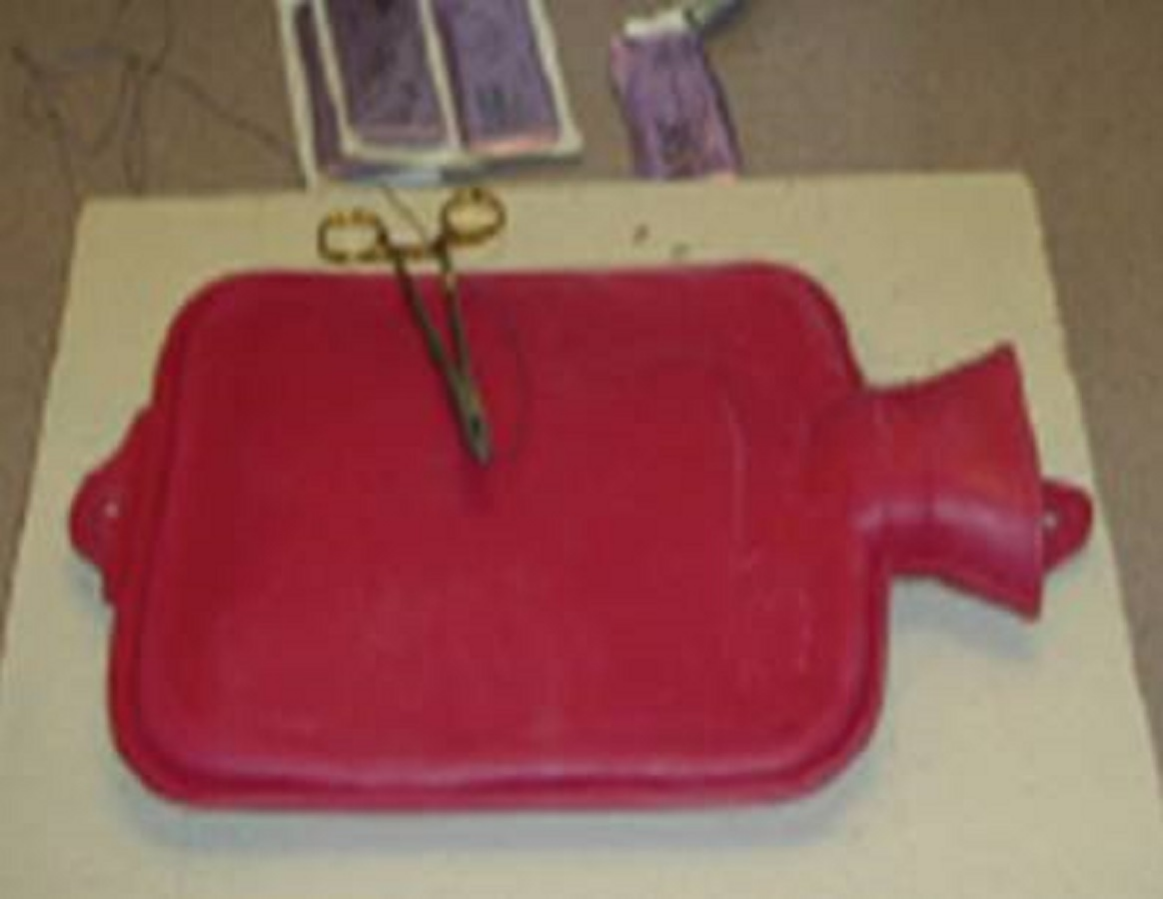
Hot water bottle model [14].

A review of literature yielded similar uterine models to the one discussed above: a knitted wool model (see Figure 5), a women’s pantyhose stocking filled with cotton wool/fiber fill (see Figure 6), a model made using two face cloths (See Figure 7), and finally a model using a hot water bottle mounted on a wooden board (Figure 8). They all have been described as low-cost learning tools to train students on using uterine compression sutures [11-14]. None specifically discuss the reliability and validity of the simulator itself. Only one of these models (the hot water bottle) was used as part of an assessment tool, and thus had some performance metrics outlined in the form of a global rating scale (GRS) and task-specific checklist [14]. Both will be detailed in the next section.

Implementation

Postpartum hemorrhage is an obstetrical emergency, and every Ob/Gyn resident must master its management. This model can be implemented in several ways. A number of courses such as Managing Obstetrical Risk Efficiently (MORE-OB) and Advances in Labour and Risk Management (ALARM) have already integrated PPH into their curriculum and are being used by obstetrical health professionals all over Canada [15-16]. Some centers consolidate the knowledge gained in these courses through team-based simulation. 

The uterine towel model for compression sutures can be added as part of the PPH module of these courses. This simulator can also be integrated into the Ob/Gyn residency curriculum as a simple task trainer during protected teaching time. This was done last year with the McGill Ob/Gyn residents, but the tool was not evaluated and there was no associated assessment of the residents. The simulator can also be integrated into a hybrid PPH simulation scenario as part of a larger assessment for the management of PPH, involving the entire team. The latter has been done at the University of Ottawa in the past: a resident had to manage PPH after a vaginal delivery in a hybrid model with a standardized patient and pelvis. They had to demonstrate knowledge and the application of medical management of PPH. The scenario continued with the need for a Bakri balloon for tamponade, of which the placement was demonstrated. The patient continued to bleed, thus the need for surgical management was identified, and the different techniques including uterine compression sutures were stated. The resident then had to demonstrate the correct placement of uterine compression sutures on a similar towel model. An objective structured assessment of technical skills (OSAT)-style PPH assessment scenario for residents is described in the section below by Quinn et al. [14]. The key to learning the correct technique is feedback “in” or “on action” and deliberate practice, or "expert-level" practice. Each resident can potentially have their own personal model to take home and practice on for self-learning until they are comfortable with the techniques. This low cost, mobile, feasible simulator can be used both as a formative or summative assessment of the residents’ surgical skill in using uterine compression sutures.

Assessment tool

There are a number of studies that show the positive effects of simulated team-based obstetrical emergency drills, including those for PPH [17-18]. Furthermore, the MORE-OB Program, which includes didactic lessons, workshops, and in-hospital simulation drills on PPH, is being implemented in hospitals on a national level [15]. One study, which looked at teaching surgical management of PPH to residents on cadavers, showed positive results in terms of improved theoretical knowledge, technical skills, and self-confidence, based on pretest and post-test questionnaires [19]. Thus, this study did not rely on any predetermined assessment tools.

**Figure 9:**
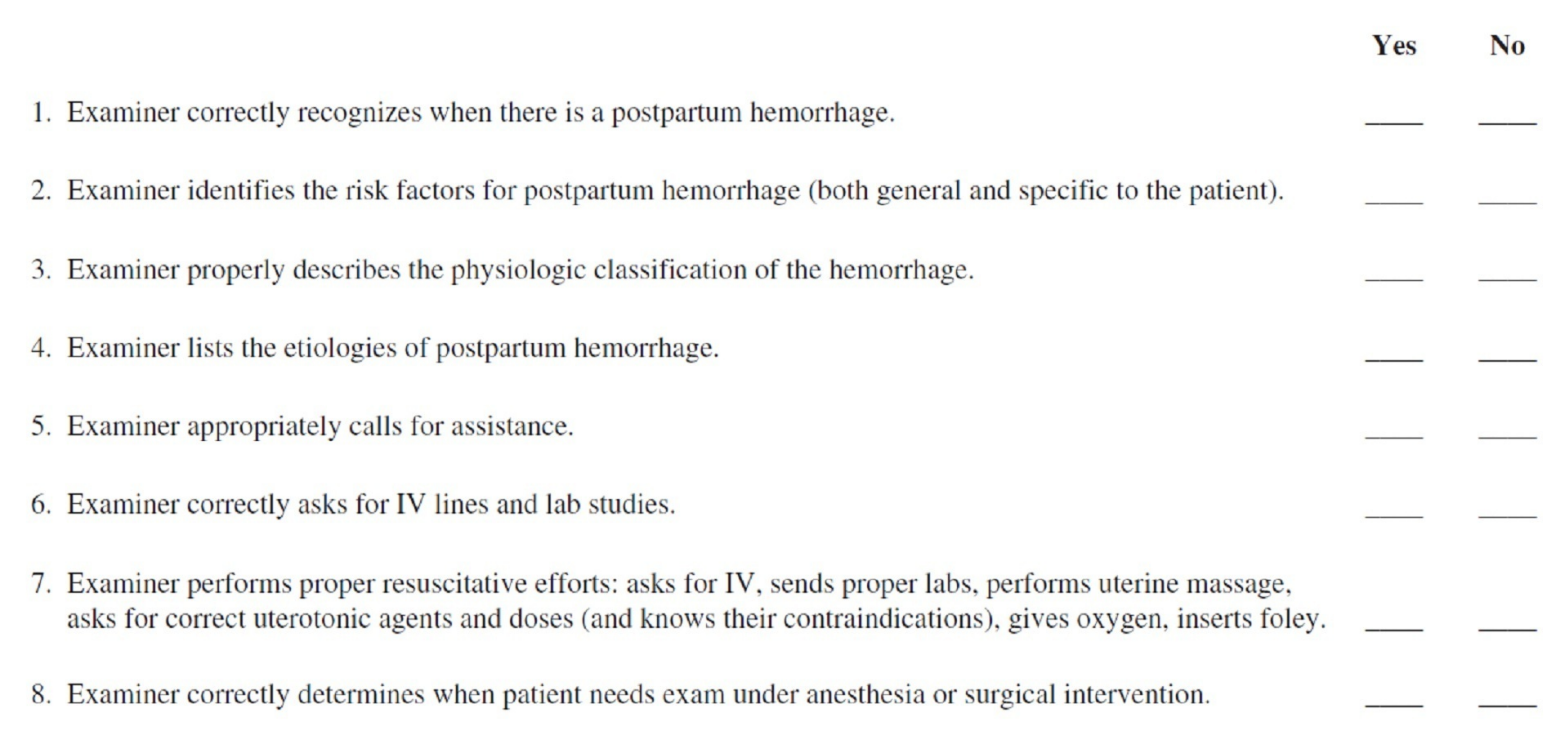
Task-specific checklist for uterine compression sutures [14].

**Figure 10:**
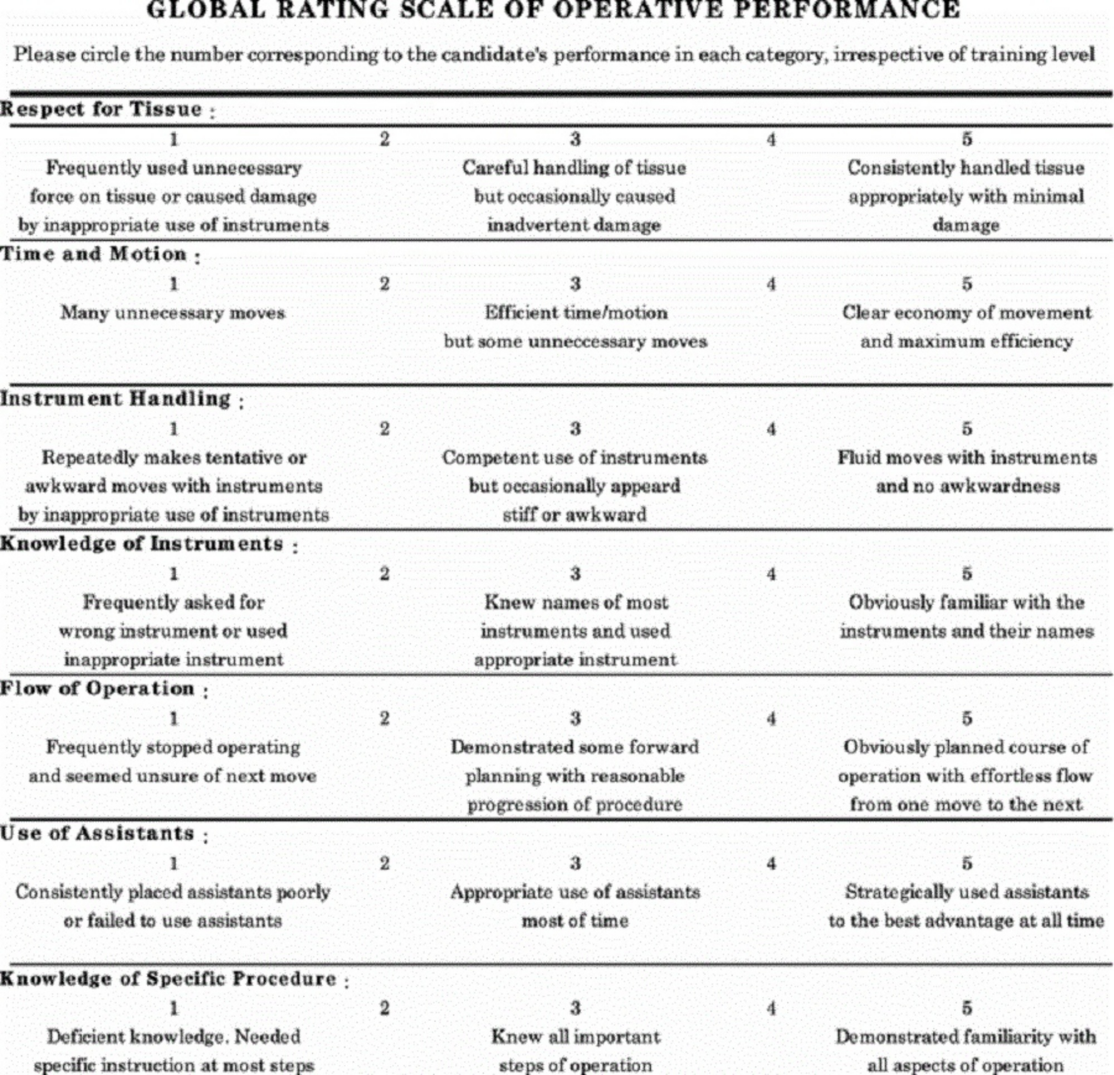
Global rating scale for surgical skills [20].

Only one article was found that evaluated a curriculum to teach and evaluate resident skills for both medical and surgical management of PPH. It describes a four-station OSAT, including a GRS and a task-specific checklist for the uterine compression suture station [14]. Instructions were provided to the residents in the form of a self-study packet and included procedural knowledge for the performance of various compression sutures. This particular study found an inter-rater reliability of 0.93 for the seven-point uterine suture task-specific checklist (See Figure 9) and 0.77 for the GRS. Upon looking at the items on this checklist, it was found that some items are vague, such as “correctly performed the steps.” These steps should be outlined in detail to differentiate the performance of different residents. The same GRS they used had been previously studied by Reznick et al. in the field of General Surgery [20]; thus, its validity in this particular Ob/Gyn setting is questionable as some of the items on the scale, such as tissue handling, cannot be adequately assessed on a plastic (or towel) model compared to a real uterus. The GRS can be found in Figure 10. Construct validity was also determined to be significant and solely based on the fact that senior residents performed superiorly compared to junior residents on both the GRS (p = 0.006) and the checklist (p = 0.04). However, as will be mentioned in the discussion below, construct validity assessment has many more intricate levels. Based on a Likert scale, residents felt that this curriculum improved both their knowledge (4.8/5) and their ability to perform uterine compression sutures (4.1/5). Moreover, a pre- and post-test showed a significant improvement of mean scores in knowledge. These outcomes measure Kirkpatrick levels 1 (Reaction) and 2 (Knowledge) [21]. No study up to date has looked at the outcomes of a similar PPH curriculum related to the performance of residents outside of simulation, in a real-world operating room, or at an even higher level, at patient outcomes (Kirkpatrick levels 3 and 4).

The rubric described in this article was developed for the formative performance-based assessment of B-Lynch, Hayman, and Cho compression sutures (See Tables 1-2). Ideally, a consensus group of experts or a Delphi survey could be used to design such a rubric; however, for our purposes, the rubric was designed using current literature and personal experience. Similarly, a GRS or a checklist could be used, such as the one described in the study above. In this case, due to the formative nature of the assessment, it was preferred to use a rubric based on the level of performance (Dreyfus levels of skills acquisition) in order to identify the resident’s expected and observed level of performance for their learning and improvement. We did not use the predetermined checklist in Figure 9 because, as mentioned previously, it was felt to be too vague and the authors did not provide information on how this checklist was developed.

**Table 1:** Rubric for performance-based assessment of B-Lynch, Hayman, and Cho uterine compression sutures – instructions for trainee

For this assessment, the trainee:
1. Recognizes when surgical management of postpartum hemorrhage (PPH) is needed.
- For uterine atony, after failed medical management or balloon tamponade
2. Knows the different options for surgical management of PPH, including the relevant anatomy.
- Uterine tamponade
- Uterine artery embolization (interventional radiology)
- Uterine compression sutures
- Uterine or internal iliac artery ligation
- Hysterectomy
3. Describes B-Lynch, Hayman, and Cho uterine compression sutures.
- What are the main differences between these techniques?
- If shown diagrams of each, can correctly them identify
4. Selects the appropriate suture/needle material and instruments for the compression suture.
- Monocryl or chromic no 1.0 or 2.0 suture (absorbable, monofilament); ideally NOT vicryl or PDS
- Either curved 70 mm (or larger) needle (B-Lynch) or straight #7 or #8 needle (Hayman/Cho)
5. Demonstrates B-Lynch, Hayman, and Cho uterine compression sutures on a uterine model.
- Clearly knows the steps and principles for each technique
- For B-Lynch/Hayman: entry/exit points are 3 cm above or below the hysterotomy incision and 3-4 cm from the lateral edge (to avoid uterine artery/ureter injury)
- For Cho: each “square” has 2-3 cm edges and, if in the lower uterine segment, is at least 3 cm from the lateral edge (to avoid uterine artery/ureter injury)
6. Performs the compression sutures in a timely and efficient manner.

**Table 2:** Rubric for performance-based assessment of B-Lynch, Hayman, and Cho uterine compression sutures – instructions for rater

Name_____________________ Stage of Residency:­­­­­_________________________ Date: ____________________­_			
Criteria	Performance levels		
	Level 3 (Competent)	Level 2 (Advanced beginner)	Level 1 (Novice)
Recognizes the need for surgical management of uterine atony based on the case presented.	Recognizes the need for surgical management, and can list all five options (balloon tamponade, embolization, internal iliac/uterine artery ligation, uterine compression sutures, and hysterectomy).	Recognizes the need for surgical management, but cannot list all five options (balloon tamponade, embolization, internal iliac/uterine artery ligation, uterine compression sutures, and hysterectomy).	Recognizes that medical management is not working and the need for something different, but does not know what the next options are.
Describes the difference between the B-Lynch, Hayman, and Cho compression suture techniques and can select the correct depiction for each.	Knows and explains the main differences: the hysterotomy is open (B-Lynch/Cho) or closed (Hayman/Cho) and B-Lynch is the only one that does not go through the anterior and posterior walls. Correctly identifies each picture.	Knows and explains the main differences, but cannot say which technique is which or does not correctly identify each picture.	Does not know the differences, thus cannot explain or identify the pictures.
Selects the appropriate suture material and instruments for compression suture.	Selects the correct suture/needle and instruments without hesitation.	Hesitates with the types of suture material and is uncertain about the selection, but correctly selects the suture/needle and instruments.	Selects the wrong suture/needle or instrument.
Demonstrates the B-Lynch compression suture technique.	Demonstrates the technique perfectly without any errors.	Most of the technique is done well, but the sutures are not 3 cm above and below the incision or not 3-4 cm from the lateral borders.	Does not know how to perform this technique (multiple errors).
Demonstrates the Hayman compression suture technique.	Demonstrates the technique perfectly without any errors.	Most of the technique is done well, but the sutures are not 3-4 cm from the lateral borders.	Does not know how to perform this technique (multiple errors).
Demonstrates the Cho compression suture technique	Demonstrates the technique perfectly and safely without any errors.	Most of the technique is done well, but the suture points are either less or more than 2-3cm away from each other, or too close to uterine vessels.	Does not know how to perform this technique. (Multiple errors).
Performs the sutures in a timely and efficient manner	Completes the suturing tasks in criteria 3,4,5 within 10 minutes.	Completes the suturing tasks in criteria 3,4,5 in more than 10 minutes.	Is unable to complete the task in the allotted time.
Overall Level of Performance: 1 2 3			

The construct validity and reliability of this assessment tool have not yet been studied, but a description of possible ways to assess both these concepts will be elaborated. Validity refers to the concept that the test measures what it is supposed to measure (what do the results or scores actually mean?) and it is the single most important characteristic of assessment data [22]. Without validity, assessment data have little to no meaning. As the goal of this assessment tool is formative/lower-stakes, the amount of validity evidence is less than that required for higher-stakes exams. Downing provides us with a simplified interpretation of Messick’s sources of test validity, which include five sources: content, response process, internal structure (includes reliability), relations to other variables, and evidence based on consequence of testing [22]. According to Downing, at least two different categories of validity evidence are required for most measures, more for higher-stakes exams. In terms of content evidence, there should be a relationship between the test content and the construct of interest. The tool’s content relates to the learning objectives for Ob/Gyn residency training, which is that: a fully trained resident must be competent to perform independently both “nonsurgical and surgical management of moderate and severe PPH”, including the use of uterine compression sutures [3]. The specific content criteria are based on current medical knowledge [1-2] as well as the described procedure in the literature by the respective authors of each technique (B-Lynch, Hayman, and Cho) [5, 9, 11], and my own personal expertise in the field of Ob/Gyn. The relationship to other variables can be assessed by comparing this new assessment tool with the checklist and GRS described in the article above as well as by discriminating between junior and senior learners (expert-novice performance comparisons). The consequences of the tool on the trainees can be assessed using a pre- and post-test questionnaire, aimed at evaluating whether the tool positively aided in their learning of the task, which is the main goal (e.g., using self-efficacy or confidence scores). Construct-irrelevant variance is a threat to validity for this specific tool and will need to be assessed. In order to decrease this threat, raters will need to be trained to use the tool in a systematic way and limit their biases. Each item in the rubric should be reviewed and deemed appropriate, in a process called item analysis (ideally as stated above by an expert group) to avoid construct under-representation, another threat to validity. The authors Quinn et al. do not explain how they came up with the items on their checklist. Adjustments to the criteria will be made if the expected level of performance is always under or over that observed. This can be done during pilot studies, before widespread implementation. Reliability is an integral part of internal structure validity and refers to the reproducibility of the data or scores of the assessment. The fewer random errors there are, the higher the reliability. The Cronbach’s alpha can be used to measure internal consistency reliability. In this case, a score of 0.70-0.79 would be acceptable as it is a lower-stakes assessment.

Eventually, this performance-based assessment of uterine compression sutures for PPH can become part of a larger higher-stakes assessment for PPH as a whole. If this becomes the case, more validity and reliability evidence will be required. For example, and similar to the study described at length, it may be integrated as part of an OSAT on PPH which would comprise several stations, including a PPH drill with a standardized patient (possibly a hybrid model), a suturing station, and a Bakri balloon insertion station, amongst other possibilities. Reliability can be increased by combining this assessment score with others, leading to a composite score, as well as by item analysis, mentioned above. Inter-rater reliability also needs to be assessed to ensure reliability. This can be done using a number of statistical methods like the Pearson correlation coefficient. Training raters to use the assessment tool will not only help decrease construct-irrelevant variance, as previously mentioned, but can also increase inter-rater reliability. Presently, the tool has only three levels of assessment, aligned with Dreyfus and Dreyfus’ levels of skills acquisition one-three: novice, advanced beginner, and competent [23]. This may be expanded to a larger scale (e.g., five) if pilot studies show any threats to reliability or validity or if it is felt that level four (proficient) and five (expert) need to be defined.

## Conclusions

Postpartum hemorrhage remains the leading cause of maternal mortality worldwide. A number of medical and surgical management techniques are available to prevent morbidity and mortality associated with PPH. Uterine compression sutures provide a more conservative surgical approach, allowing for the preservation of fertility. Ob/Gyn residents need to be adequately trained to competently perform this technique. A uterine towel model for surgical skills training in using uterine compression sutures was developed along with a performance-based assessment rubric in order to aid residents’ learning and understanding of the technique. Much work is still needed to test the validity and reliability of this tool, but based on current literature, results may be promising.
